# An approach to change the basic polymer composition of the milled *Fomes fomentarius* fruiting bodies

**DOI:** 10.1186/s40694-021-00112-9

**Published:** 2021-04-15

**Authors:** Liudmila Kalitukha

**Affiliations:** Good Feeling Products GmbH, Hansemannstr. 7, 41468 Neuss, Germany

**Keywords:** *Fomes fomentarius*, Fruiting bodies, Alkali-acidic treatment, Cell wall composition, Chitin, Chitosan, Glucans

## Abstract

**Background:**

Chitin and its derivative chitosan are readily exploited, especially in food, cosmetic, pharmaceutical, biomedical, chemical, and textile industries. The biopolymers are currently recovered from the crustacean shells after purification from the large amount of proteins and minerals. The key problems are centered around a lot of chemical waste and allergenic potential of the heat-stable remaining proteins. Fungi can be considered as an alternative eco-friendlier source of the chitin and chitosan due to the lower level of inorganic materials and absence of the allergenic proteins.

**Results:**

The work presents a new chemical assay to change the composition of the milled *Fomes fomentarius* fruiting bodies. A gradual 13-fold increase of the chitin amount accompanied by 14-fold decrease of the glucan content was obtained after repetitive alkali-acidic treatment. Raw material contained mainly chitin with 30% degree of deacetylation. After the first and second alkali treatment, the polymer was defined as chitosan with comparable amounts of N-acetyl-d-glucosamine and d-glucosamine units. The last treated samples showed an increase of the chitin amount to 80%, along with typical for the natural tinder fibers degree of deacetylation and three-dimensional fibrous hollow structure.

**Conclusions:**

A new approach allowed a gradual enrichment of the pulverized *Fomes fomentarius* fruiting bodies with chitin or chitosan, depending on the extraction conditions. High stability and fibrous structure of the fungal cell walls with a drastically increased chitin ratio let us suggest a possibility of the targeted production of the chitin-enriched fungal material biotechnologically under eco-friendly conditions.

## Background

Chitin is one of the most abundant materials in the world. The biopolymer makes up a large part of shellfish and insect exoskeletons and builds a base structure of the cell walls of some fungi [1–3].

A potential of chitin as nontoxic, biocompatible and biodegradable material is readily exploited. Chitin as well as its derivatives chitosan and glucosamine (GlcN) are used as adsorbents in filtration processes, within some wound dressings, in agriculture for plant defense and yield increase, in food processing, as cosmetic ingredient, to produce recyclable bioplastics, in the development of nanomaterials, bioadhesives, improved drug delivery systems, in medical devices etc. [2, 4, 5].

Nowadays, the biopolymer is mainly extracted from shellfish industry wastes holding up to 40% chitin. The polymer still has to be purified from the highly-concentrated proteins and calcium or magnesium carbonate (20–40% and 20–60%, respectively) usually using alkali and acids [2, 4]. Along with a process producing a lot of chemical waste, the final product is not completely free from heat-stable proteins and can cause allergic reactions in sensitized peoples. Meanwhile, allergy to shellfish is one of the most common food allergies, especially in regions with high seafood consumption [6]. To date, different fungi species are investigated as an alternative chitin/chitosan source to satisfy the expanding demand for the high-quality polymers [3–5, 7, 8].

In fruiting bodies of the higher mushrooms, the chitin amount ranged from 0.4% in *Hypsizygus tessulatus* to 30.1% dry weight in *Fomitopsis pinicola* [8–12]. Crystalline chitin microfibrils form a rigid backbone of the cell wall networked with other polymers like glucans, polyphenols and hemicellulose [1, 13, 14]. It was suggested, that in higher basidiomycetes the chitin content may be enhanced up to 95%, depending on the type of fungi and regime of treatment [14, 15].

In this work a new approach for the gradual chitin/chitosan enhancement in the powdered fruiting bodies of the wood-decay mushroom *Fomes fomentarius* was presented.

## Results

Repetitive alkali-acidic treatment of the *F. fomentarius* milled fruiting bodies led to the gradual increase of the GlcN content from 5.3% to 69% (Table 1). Separated acquisition of the acetyl groups using high-performance liquid chromatography with refractive index detection (HPLC-RI) allowed us further calculation of degree of deacetylation (DD) to understand in which form—chitin or chitosan—the polymers were presented.

**Table 1:** Glucosamine (GlcN), acetyl groups and degree of deacetylation (DD) after alkali-acidic treatment of *F. fomentarius*

Samples	GlcN, %		Acetyl groups, %		DD, %	
	Mean	SD	Mean	SD	Mean	SD
Raw material	5.30^a^	0.10	0.90^a^	0.10	27.58^ac^	4.63
#1	9.35^b^	0.05	0.95^a^	0.05	54.75^b^	2.15
#2	25.98^c^	2.38	3.40^b^	0.10	48.80^bc^	0.90
#3	69.05^d^	5.28	13.53^c^	2.35	21.38^a^	11.13

Data were obtained from three or more independent experiments, performed in triplicate and represent mean in g/100 g dw ± SD of the mean. Means in a column that do not share a superscript letter are significantly different at p < 0.05 by one-way ANOVA. See Fig. 3 for identification of the samples

Naturally, chitin is a random mix of chitin and chitosan composed of ß-(1,4)-linked N-acetyl-d-glucosamine (GlcNAc) and GlcN units. DD less as 50% points to chitin and more than 50% is characteristic for chitosan. During the study, the molar fraction of GlcN in the copolymer, measured as DD, was changed from approximately 30% in raw material to around 50% after the first and second alkali treatments (samples #1 and #2) followed by further decrease of the parameter to the end of treatment (Table 1). Thus, a proportion of chitin/chitosan was temporarily shifted toward chitosan in the middle of treatment. Otherwise the polymer was presented mainly as chitin with DD less than 50%.

**Fig. 1:**
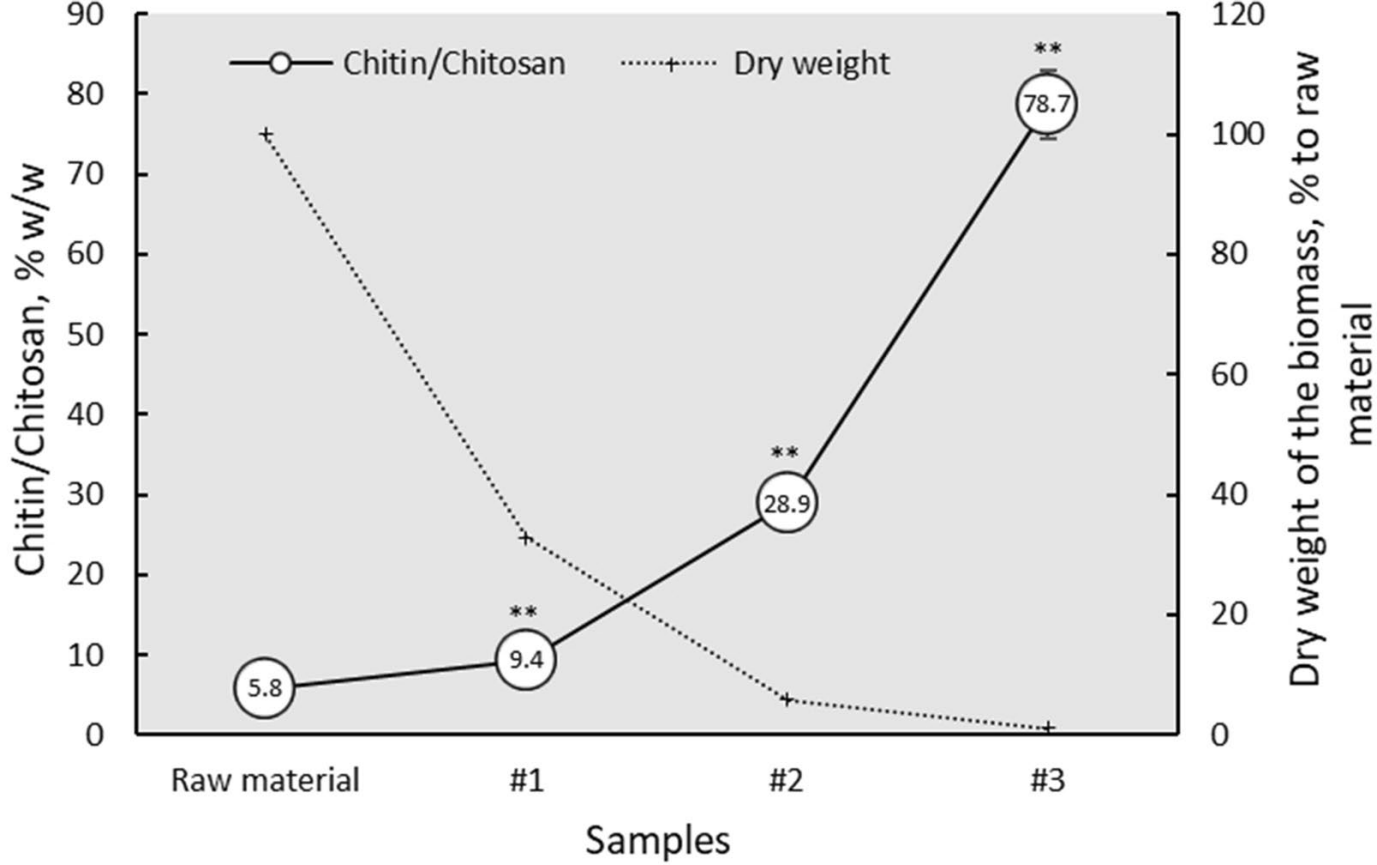
Chitin/Chitosan amount and dry weight of the *F. fomentarius* biomass within alkali-acidic treatment. The asterisks represent significant differences from raw material (P < 0.01). Means in g/100 g dw are presented). See Fig. 3 for identification of the samples

Using molar fractions of GlcN and GlcNAc, a precise calculation of the total chitin and chitosan amount in the samples was performed. Within alkali-acidic treatment of the raw material, more than 13-fold increase of the polymers was obtained, reaching as much as 80% (Fig. 1, Y1, solid line).

Fibers of the *F. fomentarius* contained naturally around 40% of the total glucans with majority of the β-glucan. The alkali-acidic treatment gradually decreased their amount until 2.5 g in 100 g dw (Table 2).

**Table 2:** Glucans in the cell walls of the *F. fomentarius* after repetitive alkali-acidic extraction

Samples	Total glucan, %		α-glucan, %		β-glucan*, %	
	Mean	SD	Mean	SD	Mean	SD
Raw material	38.48 ^a^	0.49	0.94 ^a^	0.41	37.54 ^a^	0.08
#1	35.25 ^a^	4.75	0.18 ^a^	0.05	35.07 ^a^	4.72
#2	19.42 ^b^	0.12	0.37 ^a^	0.13	19.05 ^b^	0.01
#3	2.74 ^c^	0.63	0.20 ^a^	0.12	2.54 ^c^	0.51

Data were obtained from three or more independent experiments, performed in triplicate and represent mean in g/100 g dw ± SD of the mean. Means in a column that do not share a superscript letter are significantly different at p < 0.01 by one-way ANOVA. See Fig. 3 for identification of the samples. *β-glucan values were calculated based on total glucan and α-glucan measurements

**Fig. 2:**
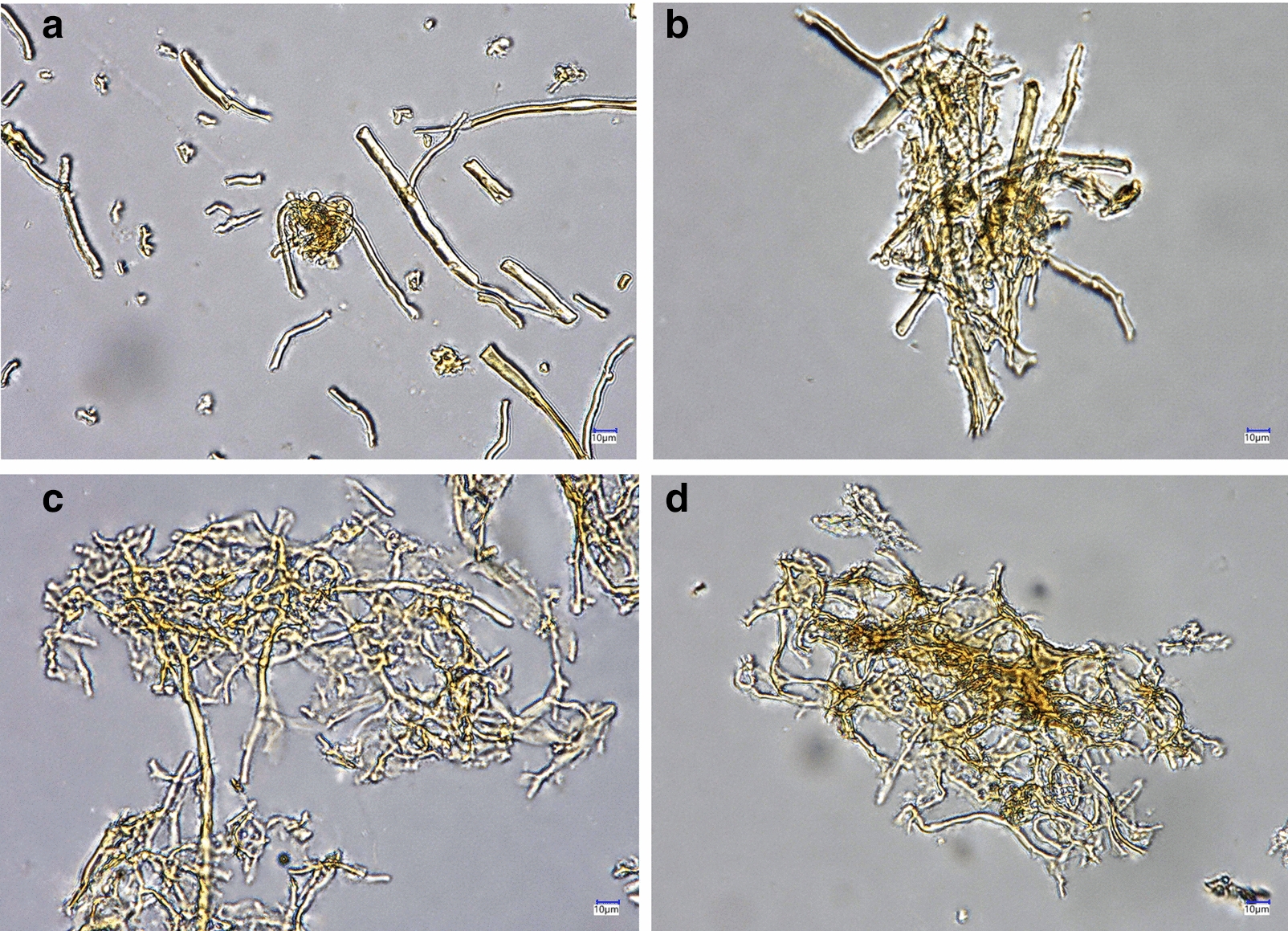
Microscopic images of the *F. fomentarius* samples after repetitive alkali-acidic treatment. **a** Raw material, **b** sample #1, **c** sample #2, **d** sample #3. See Fig. 3 for identification of the samples. Digital microscope VHX-7000 (Keyence Deutschland GmbH), transmitted light, magnification of × 667 was used

Microscopic examination of the chitin/chitosan-enriched products was fulfilled to prove whether integrity of the *F. fomentarius* cell walls was affected. Instead of the severe compositional changes, the samples showed a clear fibrous structure, identified under a digital microscope VHX-7000 (Keyence Deutschland GmbH, Neu-Isenburg, Germany) (Fig. 2).

**Fig. 3:**
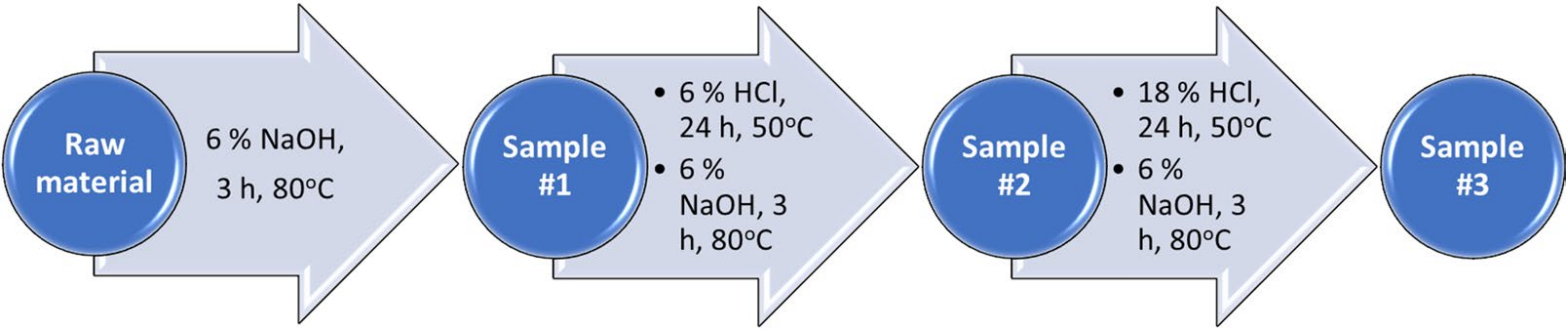
Extraction steps to change the polymer composition in the powdered *F. fomentarius* fruiting bodies. Dried *F. fomentarius* fruiting bodies were fine milled, mixed with extraction agents (NaOH or HCl) and incubated for 3 or 24 h at different temperatures as described. The liquid phase was filtered and disposed. Before changing the extraction agent, the solid phase was washed several times with demineralized hot water until neutral pH was attained. Some aliquots of the solid phase (Samples #1–3) were dried at 60 °C and used for further analysis

## Discussion

In accordance with the previously published data [12], *F. fomentarius* wild growing fruiting bodies composed of the 6% chitin (Fig. 1, raw material). Around 38% of β-glucan was measured presently, that is slightly higher than 25–32% published for tinder fungus before [12, 16]. The differences could be caused by improved hydrolysis of the cell walls after using the sulfuric acid instead of the hydrochloric one, as was recommended in the updated Megazyme protocol [17].

According to already published work, mild treatment did not lead to strong changes in composition of the main structural compounds of the *F. fomentarius* milled fruiting bodies. Chitin as well as β-glucans slightly increased (15 and 25%, respectively) in relation to the raw material [12].

It was obvious, that more severe treatment of the fruiting bodies was necessary to get a product essentially enriched in chitin or chitosan. Thus, hot alkali and acid treatments were combined consequently to wash out other cell wall compounds like glucans, polyphenols, minerals etc.

In the present work a gradual increase of the chitin or chitosan percentage accompanied by decrease of the glucan content was presented after repetitive alkali-acidic extraction. Within the treatment, described in “Methods”, the chitinous compound raised up to 13-fold, reaching a level of 80%, whereas β-glucan content decreased by factor 14 to 2.5% (Fig. 1, Table 2). Comparable high amounts of the chitinous polymers were detected earlier in isolated chitin-glucan complexes from *F. fomentarius*, *Phanerochaete sanguinea* and *Ganoderma applanatum* (72, 59 and 64–74%, respectively) after consequent extraction with different solvents like water, alcohol, benzene, alkalis and acids under varying time and temperature. The method used in this reference was unfortunately described too briefly to be reproducible [15].

Depending on the different extraction steps, the various ratios of chitin and chitosan were observed in this work. Thus, raw material contained more chitin than chitosan with a deacetylation grade of around 30% (Table 1). Comparable 26.5% DD was published for the wood decay fungus *Fomitopsis pinicola* [10]. After the first alkali treatment, the ratio chitin/chitosan was shifted toward chitosan with about 50% of deacetylation. Different from chitin, a positively charged chitosan could be soluble under certain conditions to be removed by extracting solutions. This was the case during further treatment to get 80% of the insoluble polymer. The final chitin-enriched product (sample #3) showed typical for natural tinder fibers DD of maximal 30%, thus to be defined as chitin (Table 1).

Instead of the severe compositional changes, the three-dimensional structure of the cell walls of the all samples was maintained fibrous. Microscopic images using transmitted light demonstrated hollow structure of the chitin-enriched fibers (Fig. 2d), that is common for tinder fungus (Fig. 2a) [12]. The treatment did not destroy the basic framework of the fungal cell wall, that, according to the literature, is arranged as three-dimensional crystalline chitin microfibrils [1, 13, 14]. Certain amounts of chitosan as well as more amorphous matrix like glucans etc. were washed out during treatment.

It is worth mentioning, that the economic rationale to produce chitin-enriched material from *F. fomentarius* fruiting bodies is not very promising because of the chemical waste, long and energy-consuming producing cycle and poor, lowered by the time of treatment yield of the end product. In our experiments, only around 1% of the sample with 80% chitin was recovered from the initial dry weight of the biomass (Fig. 1, Y2, dotted line), giving 0.86% of the total chitin yield in the last treated sample. In the literature, 1.3–1.4% recovery of the chitin-glucan complex after alkali-acidic extraction was mentioned for another wood-decay basidiomycete *Fomitopsis officinalis* [18], commonly known as Agarikon.

Other rationale includes rapid growing market of chitin and chitosan application, in particular in food, cosmetic, pharmaceutical, biomedical, chemical, agriculture, biotechnological and textile industries [4, 5, 9]. From 5 to 32% chitin and chitosan could be recovered from crustacean shells [19]. Among the disadvantages of the mostly used chemical methods, a lot of alkali and acid waste as well as remaining protein rests that can elicit allergic reactions in sensitized peoples should be mentioned [2, 3]. Compared to shellfish-derived polymers, the fungal ones have close to zero amount of allergic proteins, less minerals and therefore pose a potential for use in the areas of personal care, food and biomedicine [3–5, 7, 8]. Especially attractive should be fungal waste processing or biotechnological cultivation of the chitin-enriched fungal strains.

## Conclusions

A huge potential of chitin and its derivative chitosan is readily exploited. The biopolymers are used in food, cosmetic, pharmaceutical, biomedical, chemical, agricultural and textile industries. Nowadays, chitin and chitosan are mostly recovered from the crustacean shells after obligatory deproteinization and decalcification steps. Along with expensive manufacturing accompanied by a lot of chemical waste, the end product can be often contaminated with the remaining heat-stable proteins that cause allergic reactions in sensitized peoples. Fungi are considered to become an alternative source of the chitin and chitosan due to the reduced minerals and absence of the allergenic substances.

In the present work a new chemical approach to change the basic polymer composition of the milled *F. fomentarius* fruiting bodies was presented (Fig. 3, “Methods”). Up to 13-fold enrichment of the fungal biomass with chitin and chitosan was monitored after repetitive alkali-acidic treatment, depending on the extraction conditions (Fig. 1). A gradual increase of the chitin and chitosan from 6 to 80% was accompanied by 14-fold decrease of the total- and β-glucans (Table 2). Raw material contained mainly chitin with 30% degree of deacetylation. After the first and second alkali treatments, the polymer was defined as chitosan with comparable proportion of N-acetyl-d-glucosamine and d-glucosamine units. The last treated samples showed an increase of the chitin amount to 80%, along with typical for the natural tinder fibers degree of deacetylation and three-dimensional fibrous hollow structure. High stability and fibrous structure of the severe treated tinder fungus cell walls let us suggest a possibility for biotechnological production of chitin-enriched fungal material to overcome problems with chemical waste and low yield of the end product.

## Methods

### Identification of species

The wild growing fruiting bodies of the wood-destroying tinder fungus (*Fomes fomentarius* (L.) Fr.) were identified morphologically and by sequencing the nuclear ribosomal internal transcribed spacer (ITS) region. Molecular identification was performed by Alvalab molecular analysis service (LA Rochela, Spain) [20].

### Assay to change the polymer composition in the milled *Fomes fomentarius* fruiting bodies

An approach to change the polymer composition in the *F. fomentarius* isolated cell walls was based on the extraction methods published previously for different fungi [15, 21–25]. A large amount of preliminary studies using different solvents, temperature, time and ways of treatment was performed to test suitable conditions, but not included in the present work.

Dried *F. fomentarius* fruiting bodies were fine milled to obtain fibers of an average length of 10 to 50 µm, mixed 1:10 w/w with extraction agents (NaOH or HCl) and incubated for 3 or 24 h at different temperatures as described (Fig. 3). The liquid phase was filtered and disposed. Before changing the extraction agent, the solid phase was washed several times with demineralized hot water until neutral pH was attained. Some aliquots of the solid phase (Samples #1–3) were dried at 60 °C and used for further analysis.

### Chitin and chitosan measurement

The chitin and chitosan were quantified after hydrolysis with 6 N HCl (Merck, Darmstadt, Germany) for 7 h at 100 °C [26] followed by determination of the resulting GlcN using high-performance anion exchange chromatography coupled with pulsed amperometric detection (HPAE-PAD). Briefly, an ICS-5000 system (Dionex, Sunnyvale, CA, USA) equipped with a CarboPac-PA20 guard column (3 × 30 mm) and a CarboPac-PA20 analytical column (3 × 150 mm) was used for HPAE-PAD. Detection was accomplished using integrated pulsed amperometric detection (IPAD) with a gold working electrode and an Ag/AgCl reference electrode. A standard carbohydrate quadruple potential waveform was used. System controlling and data processing were performed using a Chromeleon 7.2 SR5 software. Elution was carried out with NaOH (Merck, Darmstadt, Germany) at a flow rate of 0.4 mL/min; 0.1 mL aliquots was injected. The concentration of the GlcN in the samples was calculated using an internal standard calibration method.

The amount of chitin/chitosan was calculated as a sum of the molar fraction of GlcNAc and the molar fraction of GlcN according to the following equation:

$$\begin{aligned} {\text{Chitin}}/{\text{chitosan}}, \% & = {\text{Acetyl}} \times 203.19/42 + ({\text{GlcN}} - {\text{Acetyl}} \times 203.19/42 \times 179.17/203.19) \times 161.16/179.17 \\ & = {\text{Acetyl}} + {\text{GlcN}} \times 0.9 \end{aligned}$$

where Acetyl—acetyl groups, %, measured by HPLC-RI, GlcN—total GlcN, %, measured by HPAE-PAD, 203.19—molar mass of acetylated unit, g/mol, 161.16—molar mass of deacetylated unit, g/mol, 179.17—molar mass of GlcN, g/mol, 42—molecular mass of acetyl, Da.

### Estimation of acetyl groups and degree of deacetylation

Acid hydrolysis with sulfuric acid, according to NREL/TP-510–42,618 [27], followed by HPLC-RI was used to estimate the amount of the acetyl groups. Briefly, the Series 200 high-performance liquid chromatography system with refractive index detector (PerkinElmer Life and analytical Sciences, CT, USA), equipped with Phenomenex® Rezex™ ROA-Organic Acid H + (8%), column (300 × 7.8 mm) and TotalChrom 6.3.1 software (PerkinElmer Life and analytical Sciences, CT, USA) was used. Elution was carried out with 5 mN sulfuric acid at a flow rate of 0.6 mL/min and column temperature of 65 °C. The analyte concentrations were calculated using an internal standard calibration method.

Degree of deacetylation (DD) was defined as the GlcN molar fraction in the copolymer and represented by the following equation [3]:

$$DD = 100\frac{{n_{{GlcN}} }}{{n_{{GlcN}} + {n_{{GlcNAc}} }}}$$

where n_GlcN_—the average number of d-glucosamine units, n_GlcNAc_—the average number of N-acetyl- d-glucosamine units.

### Glucan measurements

The total-, α- and β-glucans were determined using an enzyme-based assay developed for mushrooms and yeasts (Megazyme International Ireland Ltd., Bray, Country Wicklow, Ireland) according to the updated protocol from 2019 [17]. For total glucan determination, the samples (90 mg) were mixed with 2 mL of ice-cold 12 M sulfuric acid and incubated in an ice-water bath for 2 h. After adding 10 mL of distilled water, the samples were incubated in a boiling water bath for 2 h. After a neutralization step with 6 mL of 8 M NaOH, the samples were adjusted to 100 mL with sodium acetate buffer (200 mM, pH 4.5). 0.1 mL aliquots were incubated with exo-1.3-β-glucanase (20 U/mL) and β-glucosidase (4 U/mL) at 40 °C for 60 min. Then, 3 mL of glucose oxidase/peroxidase (GOPOD) was added to each tube and incubated at 40 °C for 20 min.

For α-glucan determination, 100 mg samples were stirred with 2 mL of 1.7 M NaOH for 20 min on ice. After adding 8 mL of sodium acetate buffer (1.2 M, pH 3.8) and 0.2 mL of invertase-amyloglucosidase mix (1630 U/mL and 500 U/mL), the samples were incubated for 30 min in a water bath at 40 °C. 0.1 mL aliquots were mixed with 0.1 mL of sodium acetate buffer (200 mM, pH 4.5) and 3 mL of GOPOD and incubated for 20 min at 40 °C. A yeast standard and an internal mushroom powder standard were used for control. All samples were measured at 510 nm using BioSpectrometer (Eppendorf, Wesseling, Germany) against a reagent blank. The β-glucan content was determined by subtracting the α-glucans from the total glucans.

### Statistical analysis

Data were obtained from three or more independent experiments, performed in triplicate and represent mean in g/100 g dw ± SD of the mean. Data analysis was performed using Microsoft Excel. Statistical differences were evaluated through one-way ANOVA with a confidence levels of 95% (P < 0.05) or 99% (P < 0.01).

## Data Availability

Data sharing is not applicable to this article as no datasets were generated or analyzed during the current study.

## Abbreviations

DD: Degree of deacetylation
dw: Dry weight
*F. fomentarius*: *Fomes fomentarius*
GlcN: d-glucosamine
GlcNAc: N-acetyl-d-glucosamine
GOPOD: Glucose oxidase/peroxidase
HCL: Hydrochloric acid
HPAE-PAD: High-performance anion exchange chromatography coupled with pulsed amperometric detection
HPLC-RI: High-performance liquid chromatography with refractive index detection
NaOH: Sodium hydroxide
SD: Standard deviation
